# Identification of Prognostic Immune Genes in Bladder Urothelial Carcinoma

**DOI:** 10.1155/2020/7510120

**Published:** 2020-01-20

**Authors:** Qisheng Su, Yan Sun, Zunni Zhang, Zheng Yang, Yuling Qiu, Xiaohong Li, Wuning Mo

**Affiliations:** ^1^Department of Clinical Laboratory, First Affiliated Hospital of Guangxi Medical University, Nanning, Guangxi Zhuang Autonomous Region, China; ^2^Department of Urology, First Affiliated Hospital of Guangxi Medical University, Nanning, Guangxi Zhuang Autonomous Region, China

## Abstract

**Background:**

The aim of this study is to identify possible prognostic-related immune genes in bladder urothelial carcinoma and to try to predict the prognosis of bladder urothelial carcinoma based on these genes.

**Methods:**

The Cancer Genome Atlas (TCGA) expression profile data and corresponding clinical traits were obtained. Differential gene analysis was performed using R software. Reactome was used to analyze the pathway of immune gene participation. The differentially expressed transcription factors and differentially expressed immune-related genes were extracted from the obtained list of differentially expressed genes, and the transcription factor-immune gene network was constructed. To analyze the relationship between immune genes and clinical traits of bladder urothelial carcinoma, a multifactor Cox proportional hazards regression model based on the expression of immune genes was established and validated.

**Results:**

Fifty-eight immune genes were identified to be associated with the prognosis of bladder urothelial carcinoma. These genes were enriched in Cytokine Signaling in Immune System, Signaling by Receptor Tyrosine Kinases, Interferon alpha/beta signaling, and other immune related pathways. Transcription factor-immune gene regulatory network was established, and EBF1, IRF4, SOX17, MEF2C, NFATC1, STAT1, ANXA6, SLIT2, and IGF1 were screened as hub genes in the network. The model calculated by the expression of 16 immune genes showed a good survival prediction ability (*p* < 0.05 and AUC = 0.778).

**Conclusion:**

A transcription factor-immune gene regulatory network related to the prognosis of bladder urothelial carcinoma was established. EBF1, IRF4, SOX17, MEF2C, NFATC1, STAT1, ANXA6, SLIT2, and IGF1 were identified as hub genes in the network. The proportional hazards regression model constructed by 16 immune genes shows a good predictive ability for the prognosis of bladder urothelial carcinoma.

## 1. Introduction

As the most common urological malignancy, bladder urothelial carcinoma has a complex biological behavior and is known for its high recurrence rate and easy metastasis [1]. The pathogenesis of bladder urothelial carcinoma is closely related to age, sex, smoking, chemical contact, and schistosomiasis infection [2, 3]. It involves a large number of gene expression, dysfunction, and changes of multiple signaling pathways.

At present, there are still great deficiencies in the diagnosis and treatment of bladder urothelial carcinoma. The treatment plan and prognosis of patients with bladder urothelial carcinoma are related to the presence or absence of myometrial invasion. The prognosis is good in the absence of myometrial invasion, and the degree of malignancy is high after the infiltration of the muscle layer [4]. For the latter, the standard treatment is radical cystectomy plus pelvic lymphadenectomy, but the prognosis is poor, and the choice of patients is very limited [1, 4, 5]. In recent years, the role of immunity in the development of tumors has gradually been discovered. Clinical trials of immunotherapy for tumors have achieved satisfactory results. A variety of PD-1/PD-L1 inhibitors, such as atezolizumab, have been used for first-line and second-line treatment of locally advanced/metastatic urothelial cancer [6, 7].

We believe that the analysis of immune genes may contribute to better treatment of bladder urothelial carcinoma in the future and predict the prognosis of patients. Therefore, it is necessary to explore the changes of the immune system and immune-related genes in bladder urothelial carcinoma and their roles. This study analyzed gene expression data of bladder urothelial cancer from the cancer genome tlas (TCGA). The transcription factor-immune gene network was constructed for identifying important transcription factors that regulate the immunity in bladder urothelial cancer, and a Cox proportional regression model was established based on prognosis-related immune genes. We hope to explore the potential role of immune genes in the diagnosis, treatment, and prognosis of bladder urothelial carcinoma.

## 2. Materials and Methods

### 2.1. The Expression Data of Bladder Urothelial Carcinoma from TCGA Were Analyzed

The mRNA sequencing expression data and clinical sample information of bladder urothelial cancer standardized by Fragments Per Kilobase per Million (FPKM) were obtained from the Genomic Data Commons (GDC, http://portal.gdc.cancer.gov). As of September 2019, the data of bladder urothelial cancer included 19 normal samples and 414 samples of bladder urothelial cancer. The data of TCGA can be obtained publicly without the approval of the ethics committee. Differential gene analysis of expression profile data was performed used Wilcoxon rank test by R 3.5.3.

### 2.2. Extraction of Differentially Expressed Transcription Factors and Immune Genes

The transcription factor list was obtained from Tumor IMmune Estimation Resource (TIMER, http://cistrome.dfci.harvard.edu/TIMER/) [8], and the immune gene list was obtained from ImmPort (https://immport.niaid.nih.gov) [9]. As of September 2019, 318 transcription factors and 2498 immune-related genes were included. Differentially expressed transcription factors and immune genes in bladder urothelial carcinoma were extracted from documents obtained from previous differential gene analysis. R-package “pheatmap” was used to map differentially expressed transcription factors and immune genes to show their expression in cancer and normal tissues.

### 2.3. Identification of Prognostic-Related Immune Genes and Construction of Transcription Factor-Immune Gene Network

Survival status and survival time were extracted from the clinical traits corresponding to the expression data of mRNA. The immune genes associated with the prognosis of bladder urothelial carcinoma were screened by using the single factor Cox regression model of differentially expressed immune genes with R software package “survival.” Differential immune genes were introduced into reactome database (version 70, https://reactome.org/) [10] for pathway enrichment analysis. Then, we extracted the expression of transcription factors and analyzed the correlation between the expression of immune genes and prognosis and predicted the regulatory relationship between transcription factors and immune genes according to the results of correlation analysis. Cytoscape 3.6.1 was used to construct a transcription factor-prognosis-related immune gene regulatory network. CytoHubba [11] was used to analyze the network and find the hub genes in the network.

### 2.4. Construction of Multivariate Cox Model Based on Prognostic-Related Immune Genes

R package “glmnet” was used to calculate lasso regression to narrow the range of variables, and the cross-validation method was used to find the most suitable penalty coefficient (*λ* value). The prognosis-related immune genes were included in the multifactor Cox hazard model through R package “survival,” and the survival curve and time-dependent ROC curve were drawn.

## 3. Results

### 3.1. Differential Gene Analysis

The absolute value of fold change (Log 2 transformed) was greater than 1, and *p* value was less than 0.05 as screening criterion. A total of 4876 differentially expressed genes were identified, of which 3453 were upregulated and 1423 were downregulated. The expression of immune-related genes and transcription factors were also extracted. Among them, 160 differentially expressed immune genes, 20 highly expressed immune genes, 140 low expressed immune genes, and 77 differentially expressed transcription factors were identified, of which 41 were highly expressed and 36 were lowly expressed.

### 3.2. Prognostic-Related Immune Genes Were Identified

**Figure 1:**
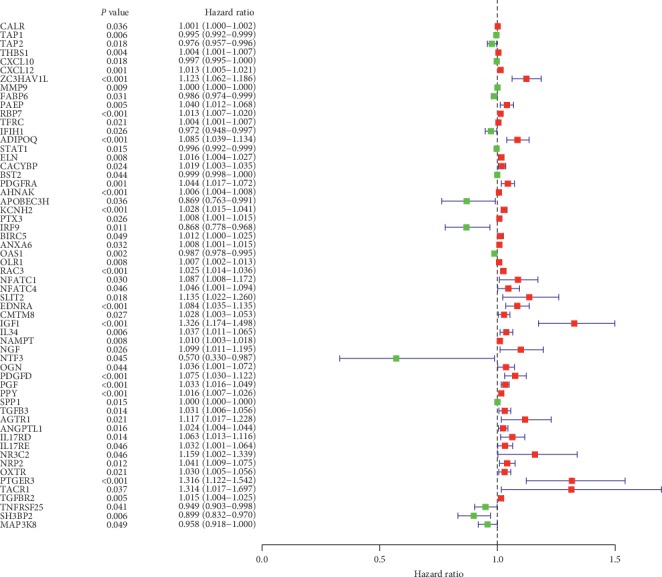
Forest plot displays immune genes affecting the prognosis of patients with bladder urothelial carcinoma (*p* < 0.01).

We obtained 414 clinical traits of bladder urothelial carcinoma, but the information is incomplete. After the missing data were omitted, 407 samples of bladder urothelial carcinoma remained. Univariate Cox regression analysis was used to preliminarily screen prognostic genes in immune genes. Finally, 58 prognostic genes were retained (*p* < 0.01, Figure 1), of which 16 were protective genes and 42 were suggestive of poor prognosis.

### 3.3. Pathway Enrichment Analysis of Prognostic-Related Immune Genes

Fifty-eight immune genes related to prognosis were introduced into Reactome database for pathway enrichment analysis. The top ten items enriched are Cytokine Signaling in Immune System, Signaling by Receptor Tyrosine Kinases, Interferon alpha/beta signaling, Signaling by PDGF, Peptide ligand-binding receptors, Signaling by Interleukins, Interferon Signaling, Formation of the Editor some, Immune System, Interleukin-4, and Interleukin-13 signaling (Table 1).

### 3.4. Transcription Factor-Prognostic-Related Immune Gene Network

**Figure 2:**
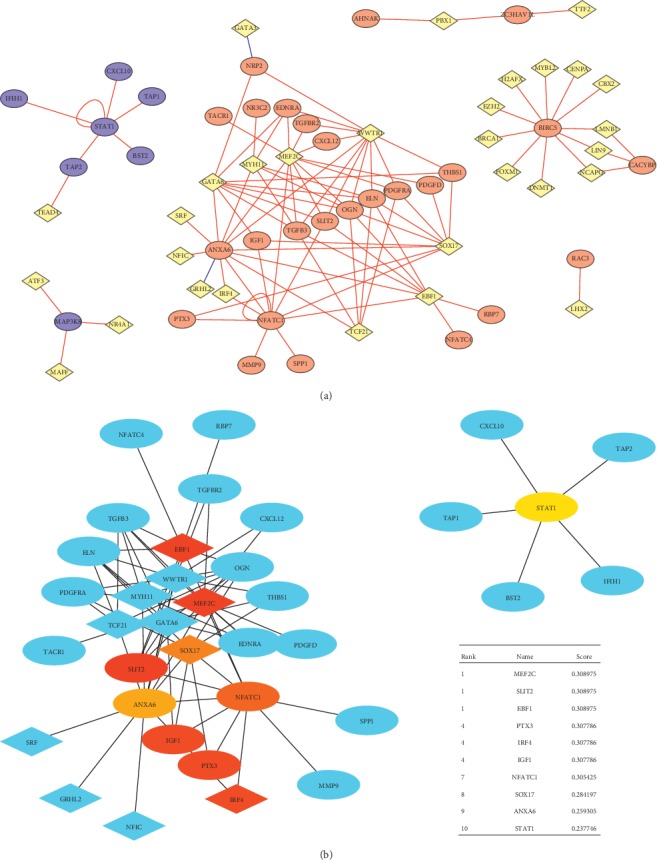
Transcription factor-prognostic-related immune gene network and hub genes. (a) Transcription factor-prognostic-related immune gene network; red round for immune genes predicting poor prognosis, blue round for predicting good prognosis one, and yellow for transcription factor and (b) hub genes were identified by CytoHubba.

We predicted the regulatory relationship between transcription factors and immune genes through the correlation of expression levels, and finally constructed a transcription factor-prognosis-related immune gene network. After screening by the Density of Maximum Neighborhood Component algorithm of CytoHubba plug-in, 10 genes were screened as hub genes in the network (Figure 2, Table 2). The transcription factors were EBF1, IRF4, SOX17, MEF2C, NFATC1, and STAT1. The immune genes included ANXA6, SLIT2, IGF1, NFATC1, and STAT1. It is worth mentioning that NFATC1 and STAT1 are both transcription factors and immune-related genes.

### 3.5. Twenty-Two Prognostic-Associated Immune Genes Were Used to Construct Prognostic Models

**Figure 3:**
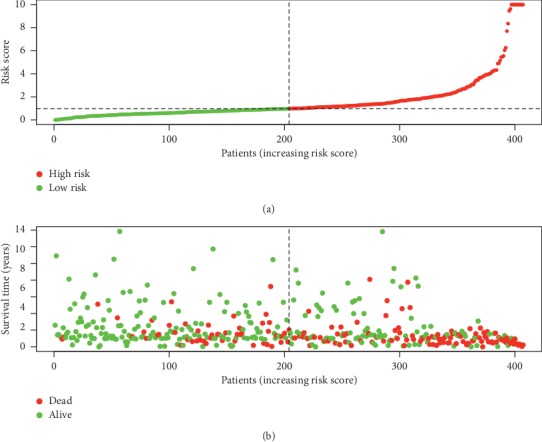
Risk score distribution and survival status of patients with bladder urothelial carcinoma.

Fifty-eight prognostic-related immune genes were screened by Lasso regression. Finally, 16 immune genes were used to construct a multivariate Cox proportional regression model (Table 3). The risk-score values of each patient were calculated. The median was used as a cut-off value to divide the experimental group into a high-risk group (*n* = 203) and low-risk group (*n* = 204). We found that the death cases were significantly concentrated in the high-risk group (Figure 3).

### 3.6. Good Predictive Ability of Prognostic Models Was Validated by Survival Analysis and Receiver Operating Characteristic Curve

**Figure 4:**
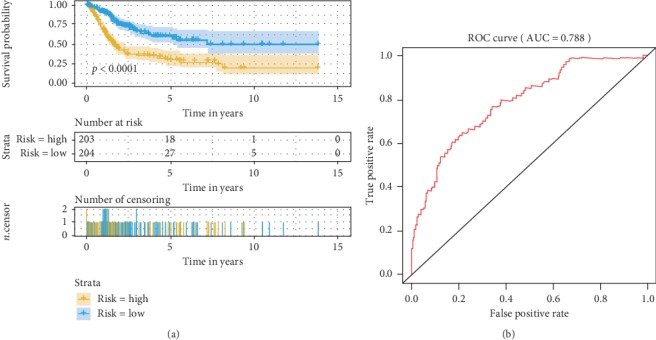
Survival curve and receiver operating characteristic (ROC) curve show that our model has good predictive ability. (a) Survival curve showed that 5-year and 10-year survival rates in the high-risk group were lower than those in the low-risk group. (b) The ROC curve showed that the prognostic model had good diagnostic efficacy.

The survival curve showed that the overall survival time of the high-risk group was significantly lower than that of the low-risk group, and the 5-year survival rate and the 10-year survival rate of the high-risk group were significantly lower than those of the low-risk group (Figure 4(a), *p* < 0.0001). The receiver operating characteristic (ROC) curve showed that the prognostic model constructed by 16 immune genes had good diagnostic efficacy (Figure 4(b), AUC = 0.788).

## 4. Discussion

In recent years, more and more attention has been paid to the changes of the immune level of bladder urothelial carcinoma. Immune targeting drugs represented by PD-1/PD-L1 inhibitors have achieved good results in the clinical treatment of bladder urothelial carcinoma [6, 7]. Tumor immunity is becoming an important link in the field of cancer diagnosis and treatment [12]. We tried to find out the factors affecting the survival of patients with bladder urothelial cancer by analyzing the expression of immune genes in bladder urothelial cancer with publicly available expression data.

In this study, a regulatory network of transcription factor-prognosis-related immune genes in bladder urothelial carcinoma was constructed. In the network, several transcription factors and immune genes were screened for hub genes. NFATC1 and STAT1 are both transcription factors and immune genes. They have been repeatedly reported in bladder urothelial carcinoma and other cancers [13–16]. Drugs targeting NFATC1 have been shown to inhibit the growth of bladder urothelial carcinoma [17, 18]. In our study, the expression of SATA1 was considered to predict a good prognosis of bladder urothelial carcinoma. Four immunogenes positively regulated by STAT1 were protective markers. This result is consistent with the current anticancer effect of STAT1 in tumors [15]. STAT1 and NFATC1 are considered upstream and downstream of PD-L1, respectively. Also, the expression of NFATC1 and STAT1 are associated with PD-L1 in bladder urothelial carcinoma [19]. Among other transcription factors, EBF1 has been reported in a variety of tumors and immune-related diseases [20–22]. DNA sequencing and qRT-PCR of urine specimens confirmed that EBF1 was different between bladder urothelial carcinoma and normal tissues [23], but this difference did not exist in upper urinary tract tumors [24], suggesting that EBF1 may be bladder-specific. Tumor markers IRF4, SOX17, and MEF2C are all involved in the development of various tumors [25–27]. In our knowledge, they have not been reported in bladder urothelial carcinoma. This study found that they may predict poor prognosis in bladder urothelial carcinoma and may regulate important immune processes. At present, most of the transcription factors in the network are related to diseases and tumors of the immune system. It is consistent with their regulatory functions on immune genes in our analysis, indicating that the transcription factor-immune gene regulatory network we constructed in this study is credible.

This study analyzed the relationship between immune genes and prognosis. Fifty-eight prognostic-related immune genes were enriched in Cytokine Signaling in Immune system, Signaling by Receptor Tyrosine Kinases, Interferon alpha/beta signaling, Signaling by PDGF, Peptide ligand-binding receptors, Signaling by Interleukins, Interferon Signaling, Formation of the Editosome, Immune System, Interleukin-4, Interleukin-13 signaling, and other pathways. Although the immune process involves multiple pathways and molecules, the results of this study show that there is a part of immune pathways related to survival time of patients with bladder urothelial carcinoma. Changes of these immune pathways may be the reason of influencing the survival time of patients. These pathways may play a role in bladder urothelial cancer and thus affect the survival time of patients. Drugs targeting key molecules in these pathways may improve the prognosis of patients.

Among the immune genes screened as hub gene, IGF1 is a growth-promoting polypeptide, which is related to the prognosis of many tumors [28]. Zhao et al. found that IGF1 in plasma of patients with urothelial carcinoma of bladder was higher than that of normal controls, and IGF1 expression was associated with an increased risk [29], but studies by Crystal Lin et al. showed that there was no correlation between IGF1 expression in peripheral blood with risk [30]. Our study based on high throughput sequencing of TCGA-derived bladder cancer tissue predicts that it may be a potential prognostic factor for bladder urothelial carcinoma. Therefore, whether IGF-1 is a potential prognostic factor in bladder urothelial cancer needs further experimental study. It is noteworthy that the receptor of IGF1 is regarded as a classical cancer-promoting molecule in bladder urothelial carcinoma [31, 32]. The SLIT2/ROBO pathway has both beneficial and harmful effects on the growth of malignant tumor cells [33]. Zhu et al. reported that SLIT2 promoter hypermethylation in bladder urothelial carcinoma and its expression in bladder urothelial carcinoma were lower than those in adjacent samples, which is contrary to the results of our study [34]. ANXA6 is a member of the annexin family. It is highly expressed in acute myeloid leukemia, pancreatic ductal adenocarcinoma, and hepatocellular carcinoma, and is associated with adverse prognosis [35–38]. We found that ANXA6 may play the same role in bladder urothelial carcinoma.

After screening for correlation of prognostic traits and Lasso regression, we finally constructed a Cox regression model for 16 immune genes related to prognosis of patients. We conducted survival analysis and plotted the working curve of the subjects, which confirmed that our model could distinguish patients with different prognosis, and patients with good prognosis had lower risk score calculated by our Cox model. We hope that this prognostic model can be clinically validated and play a role in the diagnosis, treatment, and prognosis of bladder urothelial carcinoma. There are still some limitations in our research, and our conclusions are drawn from the analysis of publicly expressed data. Some of the important genes we analyzed in the immune process of bladder urothelial carcinoma, some of which have been confirmed by a large number of studies, but some of them are still controversial or have not been studied in our knowledge, so the research on these genes will be the focus of our next step.

As far as we know, there is no systematic study to elucidate the relationship between immune genes and prognosis of bladder urothelial carcinoma. Based on exploring the relationship between immune genes and prognosis of bladder urothelial carcinoma, we explored possible transcription factors regulating these prognosis-related immune genes and constructed a good prognosis of bladder urothelial carcinoma. The model was analyzed by Cox regression. We hope that our research will contribute to the diagnosis, treatment, and prognosis of bladder urothelial cancer in the future.

## Figures and Tables

**Table 1:** Top 10 pathways of fifty-eight immune genes enriched.

Pathway ID	Pathway name	Entities found	*p* value
R-HSA-1280215	Cytokine signaling in immune system	22	2.75*E* − 06
R-HSA-9006934	Signaling by receptor tyrosine kinases	14	4.29*E* − 06
R-HSA-909733	Interferon alpha/beta signaling	8	1.41*E* − 05
R-HSA-186797	Signaling by PDGF	5	6.40*E* − 05
R-HSA-375276	Peptide ligand-binding receptors	7	2.61*E* − 04
R-HSA-449147	Signaling by interleukins	12	3.68*E* − 04
R-HSA-913531	Interferon signaling	9	5.17*E* − 04
R-HSA-75094	Formation of the editosome	2	0.001066
R-HSA-168256	Immune system	29	0.001134
R-HSA-6785807	Interleukin-4 and interleukin-13 signaling	6	0.0016

**Table 2:** Hub gene screening by the density of maximum neighborhood component algorithm.

Rank	Name	Score
1	MEF2C	0.308975
1	SLIT2	0.308975
1	EBF1	0.308975
4	PTX3	0.307786
4	IRF4	0.307786
4	IGF1	0.307786
7	NFATC1	0.305425
8	SOX17	0.284197
9	ANXA6	0.259305
10	STAT1	0.237746

**Table 3:** Cox proportional regression model.

Gene ID	Coef.
CALR	0.00111
CXCL10	−0.00215
PAEP	0.038036
RBP7	0.010577
TFRC	0.003208
STAT1	−0.00584
AHNAK	0.006656
OLR1	0.006137
RAC3	0.022579
EDNRA	0.080178
IGF1	0.17704
IL34	0.02724
NAMPT	0.01518
NTF3	−0.86755
PPY	0.012215
SH3BP2	−0.0647

## Data Availability

The data used in this study are from open public databases, and how to obtain them has been explained in the manuscript.
